# Personalized therapies in the cancer "omics" era

**DOI:** 10.1186/1476-4598-9-202

**Published:** 2010-07-29

**Authors:** Alberto Ocaña, Atanasio Pandiella

**Affiliations:** 1 Servicio de Oncología Médica, Complejo Hospitalario Universitario de Albacete y unidad AECC, Albacete, Spain; 2 Drug Development Program, Princess Margaret Hospital, Toronto, Canada; 3 Instituto de Biología Molecular y Celular del Cáncer, CSIC-Universidad de Salamanca, Salamanca, Spain

## Abstract

A molecular hallmark of cancer is the presence of genetic alterations in the tumoral DNA. Understanding how these alterations translate into the malignant phenotype is critical for the adequate treatment of oncologic diseases. Several cancer genome sequencing reports have uncovered the number and identity of proteins and pathways frequently altered in cancer. In this article we discuss how integration of these genomic data with other biological and proteomic studies may help in designing anticancer therapies "a la carte". An important conclusion is that next generation treatment of neoplasias must be based on rational drug combinations that target various pathways and cellular entities that sustain the survival of cancer cells.

## Review

A critical step towards defining a correct personalized anticancer therapy is the identification of the genes and pathways altered in the tumour of the patient, and the elucidation of their particular oncogenic role. The success of molecular studies in identifying potential molecular targets for therapeutic intervention is exemplified by the developments in the treatment of chronic myelogenous leukaemia (CML) [1]. This disease is characterized by the presence of the Philadelphia chromosome, created by a translocation which provokes constitutive activation of the tyrosine kinase Abl. The identification of this molecular alteration fostered the development of drugs such as Imatinib Mesylate that inhibit Abl kinase activity and successfully control the disease [2]. However, in most solid tumours multiple genetic lesions are expected to be required for tumour progression [3]. In fact, the clinical experience in the treatment of oncologic diseases indicates that combinations of drugs that act on different cellular targets demonstrate superiority over single agent-based treatments [4]. Yet, these combined treatments are mostly based on empirical trials and lack, in many instances, a biological rationale [5,6]. Given the positive experience that molecular knowledge has offered for the treatment of CML, efforts have been made to define the molecular alterations present in the DNA of distinct types of tumours. Here we will comment how novel high throughput techniques may help in finding more adequate and less toxic personalized anticancer therapies.

### Somatic alterations in the cancer genome

In designing proper anticancer therapies the clinical and the preclinical researchers face several important questions: How many genetic alterations exist in a tumour? How many of them are responsible of promoting tumour growth? How many should be targeted to eradicate the tumour? The availability of technologies that allow large scale sequencing and genomics analyses of several tumours, together with strong bioinformatics tools and functional studies are contributing to answering these questions. Recent studies on the molecular profiling of breast and colon [7,8], lung [9-11], glioblastoma [12,13] and pancreatic [14] cancers have described alterations in multiple genes and pathways important for the control of cell number in these tumours. Although this is an already known concept, the value of these studies is that by sequencing of the whole transcriptome of individual tumours the researchers have uncovered the number of mutated genes as well as their identity. In the analysis of the genomes of breast and colorectal cancers [7], between 60 and 80 mutations that alter the amino acid sequence of proteins (non-silent mutations) were detected in a single tumour (Table 1). Noteworthy, although some of the genes were overlapping, most of them were not coincident, not only when comparing breast and colon tumours, but also when comparing tumours of the same type but from distinct patients. It should however be mentioned that these transcriptomic sequencing studies, while offering important molecular information on individual tumours, must be complemented with other genomic analyses, as they may, for example, miss the overexpression of non-mutated HER2 that is present in one out of four patients with breast cancer, and that represents a relevant clinical target [15]. Another more recent study identified a larger number (292 mutations in coding regions, of which 187 were non-synonymous) of mutated genes in a cell line of colorectal cancer [16]. It is possible that these differences may stem from the fact that some works reported results from patients, while others used cell lines which may accumulate lesions along their *in vitro *establishment. On the other side, sequencing of acute myeloid leukaemia (AML) samples have identified 10 mutations in protein-coding mRNAs [17,18]. Additional massive sequencing efforts, such as those carried out by the International Cancer Genome Consortium, will help in establishing a more accurate measurement of the average mutated genes/tumour.

**Table 1:** Somatic mutations in cancer exomes and pathways affected

Tumour type (sample size)	Genes analyzed	Average number of mutated genes/patient	Major pathways deregulated	References
Breast (*n *= 11)	18,191	62	RTKs, PI3K, NFκB,	[7]

Colorectal cancer (*n *= 11)	18,191	88	RTKs, PI3K, Cell adhesion, Cytoskeleton, Extracellular matrix	[7]

Colorectal cancer (*n *= 1)	Genome	292	Transcription (SPDEF), Metalloproteases (MMP28), PI3K, BRAF	[16]

Glioblastoma (*n *= 22)	20,661	47	RTKs, PI3K, Cell cycle, DNA damage (p53), Neuronal-type pathways (ionic channels), IDH1	[13]

Pancreas (*n *= 24)	20,661	48	KRAS signalling, DNA damage control, Cell cycle, TGFβ pathway, Wnt/Notch signalling, Cell adhesion and integrin signalling, MAPK signalling, Apoptosis	[14]

Glioblastoma (*n *= 206)	601 selected genes	NA	RTKs, NF1, DNA damage, PI3K, Cell cycle, methylation, mismatch repair	[12]

Lung cancer (*n *= 188)	623 genes implicated in cancer	NA	RTKs, DNA damage control, RAS (K and N), NF1, LRP1B (lipid metabolism), MAPK signalling, Wnt signalling, STK11 (Ser/Thr kinase)	[10]

Lung cancer (*n *= 371)	NA	NA	Cell cycle, PI3K, RTKs, Tyrosine phosphatases, cAMP, Angiogenesis, NKX2-1 (pneumocyte differentiation)	[9]

Lung cancer (*n *= *1*)	Genome	134	Cell cycle (Rb), DNA damage (p53), DNA helicase CHD7	[11]

Mesothelioma (*n *= 4)	15,000	6	DNA damage, Extracellular matrix, Mitochondrial reductase activity, proteasome, Apoptosis	[54]

Diverse cancers (*n *= 210)	518 kinases	NA	RTKs, JNK, MAPK,, BRAF, DNA damage control	[20]

Renal cancer, clear cell (*n *= 101)	3544	NA	Histone modifications (SETD2, JARID1C, UTX), VHL, NF2, HIF1A, PMS1 (DNA mismatch repair), WRN and NBN (DNA double strand repair)	[55]

Diverse cancers (*n *= 3131)	NA	NA	Kinases, cell cycle, NFκB, Myc, Apoptosis, Cell adhesion, DNA methylation, microtubule organization, transcription	[22]

Acute Myeloid leukaemia ((*n *= 1)	Genome	10	NRAS, NPM1, IDH1, CDC42, IMPG2, ANKRD46, LTA4H, FREM2, CEP170	[17,18]

Ovarian granulosa cell tumours (*n *= 4)	Genome	NA	FOXL2	[56]

RTKs: receptor tyrosine kinases. NA: not available

By using bioinformatics analyses those authors indicated that most of the mutations in an individual breast or colon tumour were silent in terms of favouring tumour progression [7]. These analyses indicated that not more than 15 genes, that they term CAN genes (from "cancer" genes), are responsible for supporting tumour viability in each tumour. Some of the mutations identified affected genes that participate in the PI3K or NFκB routes, two pathways linked to cell survival/proliferation, and that are potential therapeutic targets (Table 2). An important question that must be addressed is how many of these CAN genes must be targeted for efficient therapy of a tumour. On the basis of age-incidence data, some authors proposed that genetic alterations in 5-7 CAN genes may be required for solid tumour generation [19], and it is therefore expected that targeting of these genes or some of their downstream actor proteins may be therapeutically effective. We will comment later some biological studies that in fact support the use of a restricted number of targettable proteins that fall within that latter number.

**Table 2:** Drugs in clinical development against pathways identified in genomic/proteomic studies

**Receptor Tyrosine Kinases**		
*Pan-ErbB receptors*		
CI-1033	Pfizer	phase II[57,58]
BIBW-2992	Boehringer Ingelheim	phase II[57,59]
Neratinib	Wyeth-Ayerst	phase III[60,61]
*MET*		
MK-2461	Merck	phase I/II[62]
XL184	Exelixis	phase II/III[62]
MetMAb	Genentech	phase I[63]
*FGFR*		
MK-2461	Merck	phase I/II[62]
Brivanib	BMS	phase II[64]
		
**K-RAS-RAF**		
PLX4032	Plexxikon Inc/Roche	phase I[65]
		
**PI3K-AKT Inhibitors-mTOR**		
Dual PI3K-mTOR		
BEZ235	Novartis	phase I/II[30,66,67]
XL765	Exelixis	phase I[68]
SF1126	Semafore	phase I/II[68,69]
BGT226	Novartis	phase II[68]
*PI3K Inhibitors*		
XL147	Exelixis	phase I[68]
BKM120	Novartis	phase I[68]
GDC0941	Genentech	phase I[70,71]
*AKT inhibitors*		
Perifosine	Keryx	phsae I/II[72-74]
GSK690693	GSK	phase I[75-77]
MK2206	Merck	phase I[68]
*mTOR*		
OSI027	OSI Pharmaceuticals	phase I[68]
AZD8055	AstraZeneca	phase I/II[68]
		
**MAPK inhibitors**		
*MEK Inhibitors*		
CI-1040	Pfizer	phase I/II[78,79]
AZD6244	AstraZeneca	phase I/II[80,81]
XL518	Genentech	phase I[71]
		
**Cell Cycle**		
Flavopiridol	Sanofi-aventis	phase II/III[82]
SNS-032	BMS	phase I/II[31]
R-547	Roche	Phase I/II[83,84]
Seleciclib	Cyclacel Pharmaceuticals	Phase I/II[85]
		
**Histone Deacetylase inhibitors**		
Vorinostat (SAHA)	Merck	Phase I/II[86]
Romidepsin	Gloucester Pharmaceuticals	Phase I/II[87,88]
MGCD0103	MethylGene, Inc	phase I/II[89]
LBH589	Novartis	phase I/II[90,91]
		
**Demethylating agents**		
Azacitidine	Celgene	approved[92,93]
Decitabine	Eisai Pharmaceuticals	approved[94]
		
**DNA repair**		
*PARP*		
Olaparib	KuDOS Pharmaceuticals/AstraZeneca	phase II[95]
AG-014699	Pfizer	phase II[96]
*ATM*		
KU-55933	KuDOS Pharmaceuticals	preclinical[97]
		
**Matrix Metalloproteinases**		
Neovastat	Æterna Laboratories	phase III[98,99]
Prinomastat	Pfizer	phase III[98,100,101]

Additional studies carried out in glioblastoma multiforme (GBM) [12,13] and pancreatic cancer [14], in addition to describing the somatic alterations in the DNA, advanced to more precisely defining the molecular pathways altered in these pathologies. Interestingly, in these tumours the number of mutated genes was lower (around 40 mutations/tumour) than in breast or colorectal cancer. By using similar and complementary techniques to search for point mutations, as well as gains and losses of genetic material, the two different reports analyzing the genomes of patients with GBM satisfyingly came to common conclusions [12,13]. These reports observed frequent alterations in three major signalling pathways that control cell proliferation: the receptor tyrosine kinase-PI3K route, the p53, and the retinoblastoma (RB) tumour suppressor pathways. Interestingly, some of these pathways were also found to participate in pancreatic as well as colon and breast cancer tumours, indicating overlapping of signalling pathways that may be critical in the genesis/progression of solid tumours. Interestingly also was the fact that mutations in the genome of GBM patients accumulated in patients treated with the alkylating agent temozolomide, a chemotherapeutic used in this pathology which is also highly mutagenic. Moreover, another study in which the genes coding for kinases of patients with several types of cancer were analyzed also showed that the highest prevalence of mutations in the kinases corresponded to GBM patients treated with temozolomide [20]. This indicates that resistance mechanisms may develop in these patients due to additive molecular alterations that favour the development of clones of cells resistant to the action of classical treatments. The sequencing efforts carried out in GBM also identified isocitrate dehydrogenase 1 (IDH1) as a protein that could act in the pathogenesis of this disease [13,21]. Interestingly, IDH1 was also found to be mutated in the genome of AML patients [18].

In lung cancer, using single nucleotide polymorphism (SNP) arrays, Weir et al. [9] analyzed the presence of copy-number alterations of chromosomal regions in a large proportion (n = 371) of lung tumours, and found frequent gains or losses of chromosomal regions. Some genomic alterations, such as copy number gain of chromosome 5p occurred in a high number of patients (60%). These alterations affected genes known to be frequently involved in lung cancer, such as *EGFR/HER1, CCNE1*, or *KRAS*. In addition, the paper describes a novel player in lung cancer pathophysiology, the NKX2-1 gene product, a transcription factor implicated in the formation of lung pneumocytes. Knocking down the expression of this protein in NCI-H1925 lung cancer cells decreased their ability to grow in an anchorage-dependent manner, indicating that this protein may represent a novel lung cancer promoting oncogene, and an interesting novel therapeutic target. Another report analyzed somatic mutations of 623 genes in 188 lung cancer samples [10]. These genes were selected for their already known implication in oncogenesis. *KRAS *or *EGFR *were mutated in a substantial proportion of patients; but, in addition, several other genes not formerly associated to lung cancer were identified, and included tumour suppressors (*NF1, RB, ATM*, and *APC*), as well as tyrosine kinase genes (*ERBB4*, ephrin receptor genes, *KDR, FGFR4*, and *NTRK*). Of note, 132 of the 188 tumours had at least one mutation in genes that participate in MAPK signalling. Also, mutations in multiple genes of the Wnt pathway were observed in 29 of the 188 samples. Frequent mutations were also detected in DNA damage response genes, including *TP53*, and *ATM*. This clustering of mutations in certain pathways which play a role in oncogenesis points to the possibility of interfering with them for the treatment of lung tumours. Moreover, from a more ample perspective the molecular studies mentioned above open outstanding possibilities in terms of better focusing on certain targeted therapies for these tumours, based on drugs that target components of the identified signalling pathways. Another report confirmed the relevance of p53 and Rb in lung cancer [11]. Here the researchers sequenced the whole genome of a lung cancer cell line and found 22,910 somatic mutations, of which 134 were included into coding exons, 94 of them causing changes in the primary sequence of the coded proteins.

An ample study of somatic copy number alterations performed on 3131 tumour samples, and also using high resolution SNP arrays identified a median of 12 gains and loses in each patient tumour [22]. Frequent alterations in the control of cell cycle progression, apoptosis, DNA damage control, or kinase activity were reported.

### The contribution of functional genomics to cancer therapy

A strategy that has been recently developed to massively identify potentially useful therapeutic targets is based on functional genomics studies using RNA-interference (RNAi) screenings. In one such functional screen based on the knockdown of 2,924 genes selected for their potential implication in tumour generation/progression, Schlabach et al. identified between 80-150 gene products important for tumour survival/proliferation [23]. Of them, 19 genes were shared by the three distinct tumoral cell lines analyzed (two from colon cancer, and one from breast cancer). Interestingly, the list of the identified proteins showed significant overlap between distinct tumoral cell lines, but were different from those identified to be essential for the survival of normal breast epithelial cells [23]. This indicates the existence of qualitative differences between normal and malignant cells with respect to proteins that support their respective viabilities. This is highly relevant from the therapeutic point of view, as it indicates that the targeting of proteins that specifically support survival of cancer cells is realistic, and may spare normal cells, therefore representing an efficient and likely safe therapeutic strategy. In addition, these authors also found a certain degree of overlapping between proteins that support viability in tumours from the same tissue origin. However, they also found proteins whose function was required to sustain viability of one tumour cell, but did not play a critical survival role in others. Thus, knock down of the protein phosphatase PP1 seriously affected survival of HCC1954 colon cancer cell line, but did not substantially affect DLD-1 colon cancer cells. These results support the concept of the distinct susceptibility of different tumours, and stress the importance of adequately selecting protein targets to achieve therapeutic success. Analogous functional genomic studies in haematological malignancies led to the identification of the IRF4 and genes regulated by this protein as potential therapeutic targets in multiple myeloma [24], and the demonstration that tyrosine kinases play an important role in sustaining AML vitality [25]. This last work is particularly interesting as the authors used fresh samples from patients to identify, by also using RNAi techniques, potentially relevant drug targets. More ample RNAi screening techniques, based on the knockdown of up to 17,000 different genes [26], have allowed an almost universal analysis of the participation of the proteome in tumour survival, and have identified pathways and proteins that participate in tumour proliferation/survival [27].

In addition to finding potentially useful drug targets, these RNAi-based functional genomic screens have also been used to uncover mechanisms of drug resistance. Bernards and colleagues explored proteins involved in trastuzumab resistance in breast cancer and identified PTEN as one of the principal proteins whose lack of function was linked to trastuzumab resistance [28], in agreement to previous reports [29]. Moreover, preclinical data indicated that combination of agents that target HER2 and the PI3K route reverts resistance to trastuzumab [30].

**Figure 1:**
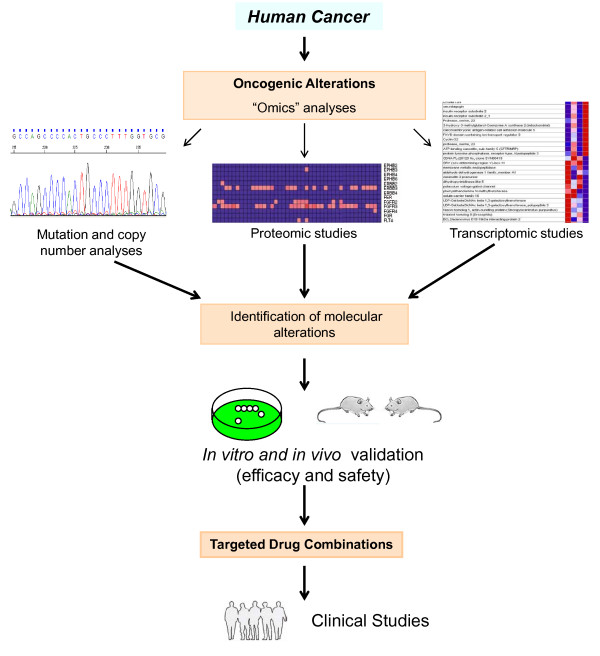
**Stepwise selection of antitumoral therapies based on individual oncogenic alterations**. Analyses of oncogenic alterations in individual tumour samples using "omics" techniques should allow the identification of candidate targets for therapeutic intervention. Experimental preclinical validation of these targets is critical in order to proceed to the clinical testing of drugs that act on the selected targets.

In spite of the value of these studies, one of the potential pitfalls is the identification of relevant targets *in vitro *whose *in vivo *manipulation could be highly toxic. It is therefore mandatory to proceed into *in vivo *testing the manipulation of those targets (Figure 1).

### Biological findings support the use of combined targeted therapies

An important aspect of the functional genomics assays is the finding of individual proteins whose targeting is deleterious for the tumoral cell, opening the door to the development of drugs that by interfering with their action may be therapeutically relevant as single agents. However, as mentioned above, the clinical experience suggests that efficient cancer treatment usually relies on the combination of agents that target distinct oncogenic networks. The fact that combination of agents may be more efficient than single agent treatment may be due to the heterogeneity of tumours, in some cases promoted by some anticancer treatments that either act as mutagens, as mentioned above in temozolomide-treated patients, or by the mutational heterogeneity within a single tumour. This heterogeneity has been recently proposed as a limit for the value of high throughput sequencing efforts in individual tumours [31]. The cross-talk between intracellular signalling pathways mainly activated by RTKs represents another reason to explain the efficacy of combination strategies. It has been reported that inhibition of certain pathways, such as the mTOR route may lead to increased MAPK activity [32]. Therefore, ablation of signals through both routes is required for efficient antitumoral action. Moreover, combined inhibition of PI3K and MAPK routes has shown superior antitumoral effect compared to individual targeting of either pathway [33].

Elegant biological studies supporting that adequate targeted drug combinations based on genomic profiling may be effective in cancer treatment has been offered by reports from the Massagué group [34,35]. These researchers used genomic and imaging techniques to analyze a particularly relevant property of tumoral cells, i.e. metastatic dissemination. Their strategy was based on the injection of a breast cancer cell line into the bloodstream of mice, and the selection of tumoral clones that homed to several organs. In their studies, genomic profiling identified signatures of genes that were particular of the cells that metastatized to a certain tissue. The researchers then selected some of these genes for further biological analyses, and suspected that four of them, Epiregulin, COX2, MMP1 and MMP2, represented therapeutic candidates in the case of lung metastases. In fact, experiments of gene knockdown confirmed the importance of these genes in breast cancer tumour growth in nude mice. Interestingly, single knockdown of only one of the coding mRNAs had little effect on the growth of these tumours as compared to double knockdowns. But the most impacting results were obtained by quadruple knockdowns. Reduction of the expression of the four mRNAs coding for the respective proteins fully abrogated tumour growth. These genomic-functional studies were complemented with targeted therapies aimed at neutralizing the activity of the four selected protein targets. Again, single agent treatment was less effective than combinations in preventing extravasation and lung colonization by the tumoral cells. Also, combination of three targeted agents, expected to neutralize the function of all four proteins, was more effective than dual combinations, and the triple combinations almost fully prevented lung invasion by the tumoral breast cancer cell population. Another recent analogous study identified a set of genes that mediate breast cancer metastasis to the brain [36]. Interestingly, some of them (COX2 and HB-EGF) share molecular identity or properties with those formerly described to participate in metastatic spreading to the lung. However, other proteins such as the sialystransferase ST6GALNAC5 was specific for cells that metastatized to the brain, and could be involved in facilitating cell passage through the blood brain barrier. The findings of Massagué and colleagues come in support of the well established clinical concept that drug combinations are superior in efficacy to single agents to treat solid tumours. The added value of their work is the use of molecular and biological tools to define and verify adequate targets for therapeutic intervention.

### Combined targeted therapies guided by proteomic studies

Probably the genomic portraits are insufficient in guiding the selection of antitumoral therapies. Indeed, studies on receptor tyrosine kinase (RTK) targeting in cancer have offered insights into the convenience of complementing the genomic data with proteomic studies to establish efficient anticancer therapies. Stommel et al. [37] analyzed the phosphotyrosine content (indicative of activation) of 42 different RTKs in GBM. This disease may present alterations of the EGFR that result in its constitutive activation. However, response rates to agents that target exclusively the EGFR are poor. Stommel et al. found consistent activation of three or more RTKs in 19 out of 20 GBM cell lines. These RTKs included the EGFR, ErbB3/HER3, platelet-derived growth factor receptor α (PDGFRα), and MET. These researchers then explored the value of targeting these activated receptors using several *in vitro *models. Inhibition of the activity of individual receptors had a marginal effect on the growth properties of the GBM cell lines. However, combination of drugs that targeted two receptors was superior to single agent treatments. Furthermore, triple combinations were more efficient than double combinations, and practically abolished tumour growth in soft agar colony forming assays. The authors conclude that coactivation of multiple RTKs may sustain cell proliferation in GBM, and that adequate treatment of this pathology must include a careful evaluation of the RTKs activated.

In line with those findings, two additional reports showed the importance of targeting multiple RTKs in breast [38] and lung cancers [39]. Both studies attempted to identify the mechanisms of resistance to therapies that target HER (Human EGFR-like Receptors) receptors. In the breast cancer study, resistance to gefitinib or erlotinib, agents that act on the EGFR/HER1, was accompanied by increased tyrosine phosphorylation of ErbB3/HER3 [38]. Similarly, gefitinib resistance in lung cancer was also found to be accompanied by ErbB3/HER3 signalling [39]. This up-regulation of ErbB3/HER3 signalling was due to amplification of MET. Concomitant treatment with gefitinib and the MET inhibitor PHA665752 provoked a decrease in cell survival not obtained by single drug treatments. The fact that multikinase inhibitors have reached the oncology clinic adds value to these experimental results and paves the way for the development of multikinase targeting strategies guided by proteomic analyses of the activation state of the kinome.

### Limitations of targeted therapies based on genomic profiling

**Figure 2:**
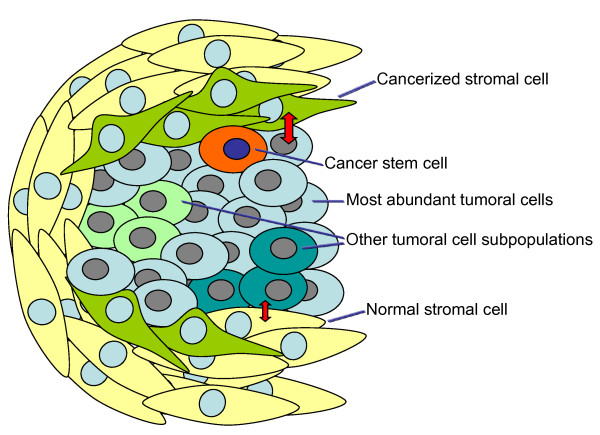
**Tumoral cell types that contribute to the heterogeneity of tumours**. In addition to the most abundant tumoral cells, the genetic instability of tumours is responsible for the establishment of genetically distinct tumoral cell subpopulations, which may escape genomic detection as they may be diluted within the tumoral mass by the most abundant tumoral cells. Another important cellular component that usually bears genomic and transcriptomic differences with respect to the majority of tumoral cells are the cancer initiating/stem cells. The stromal cells that surround or are included into the tumoral mass may provide/receive proliferation/survival signals by crosstalking with tumoral cells. In certain neoplastic diseases, the stromal cells critically contribute to the survival or dissemination of the tumoral cells, and may bear genomic alterations that favour their tumor-supporting properties. These cancerized stromal cells and the rest of the cellular components of tumours must be targetted to achieve an efficient antitumoral response.

While the above mentioned studies offer unquestionable useful information about relevant targets for therapeutic intervention in cancer, several factors that can limit the efficacy of targeted therapies must be considered. One is the presence of molecular heterogeneity within a particular tumour [40]. The well known genetic instability of tumour cells may be responsible for the generation of different subclones of tumoral cells that could be represented at different amounts in the tumoral tissue (Figure 2). Those present in higher amounts are expected to be responsible for providing the genomic information, diluting the genomic landscapes of other tumoral cells present at lower amounts. The latter therefore escape detection by those genomic/proteomic analyses and result in tumour relapse, since targeted therapies are expected to focus on alterations present in the mostly abundant cells. Exemplifying this concept is the fact that chronic exposure to gefitinib, an agent that targets the EGFR, results in emergence of resistant lung cancer cells bearing a mutation in the EGFR that renders these tumoral cells insensitive to the drug [41]. Presumably, cancer initiating/stem cells represent another source of failure to targeted treatments, as they are expected to represent a minority of the cellular constituents of the tumour. Strategies to identify drugs that target these tumour initiating cells are being developed and may represent useful additions to targeted therapies [42].

Another factor that limits the efficacy of targeted agents is the presence of parallel activation of downstream pathways. A well characterized example is the preclinical identification of the lack of antitumor activity of agents against RTKs when mutations at the K-RAS and PI3K genes were present [43,44]. Translation of these studies to the clinic confirmed lack of activity of anti-EGFR antibodies like cetuximab or panitumumab in colon cancer tumours with K-RAS mutations [45,46]. These studies have been of much importance in selection of anti-EGFR therapies in colorectal cancer, as patients whose tumours express mutated forms of K-RAS are now excluded from treatments based on anti-EGFR drugs. In addition, among colorectal tumours carrying wild-type KRAS, other additional mutations in genes participating in EGFR signalling may cause resistance to anti-EGFR therapies. Thus, mutation of BRAF or PIK3CA or loss of PTEN expression may result in resistance to EGFR-targeted monoclonal antibody treatment [47,48].

Another important tumour component that is gaining importance as a targettable cell type is the stromal compartment. In multiple myeloma, a disease characterized by the accumulation of tumoral plasma cells, the interaction of these cells with the stroma is expected to be critical for sustaining survival of the myelomatous cells, and to provide drug resistance [49]. Moreover, treatments that target both the myeloma cell and the bone marrow microenvironment, such as the proteasome inhibitor bortezomib, or the immunomodulatory agents derived from thalidomide, have shown anti-myeloma activity and have reached the myeloma clinic [50]. Interestingly, genetic studies have demonstrated the presence of genetic alterations in the bone marrow mesenchymal cells of patients with multiple myeloma, supporting the concept that the stroma in the vicinity of the tumour may be cancerized [51]. In other neoplastic pathologies such as breast cancer, mesenchymal stem cells have been shown to promote metastatic dissemination of the tumoral cells [52]. Moreover, recent studies perfomed in myelodisplastic syndromes support the concept that initial alterations in the stromal compartment may favour the generation of secondary leukemias in a mouse model, further supporting a potential role of the stroma in the pathophysiology of at least certain tumours [53]. Therefore, efficient antitumoral therapies must also take into consideration these variables in order to achieve long lasting remissions.

## Concluding remarks

The power of high scale "omics" analyses coupled with the ample portfolio of targeted drugs under clinical development and already approved offers hope for a more effective and individualized anticancer therapy. It is rewarding to observe that sophisticated genomic, proteomic and biological studies reinforce the already known clinical strategy of using drug combinations to treat cancer patients (Figure 1). The molecular and biological data herewith commented also offers clues as to how many targets, pathways or functions should be attacked. The preclinical evidence that targeting from one to four proteins may impede proliferation, survival or invasion properties of cancer cells indicates that these cells depend on a limited number of proteins to carry out these biological functions. That the studies mentioned above have correctly targeted some of these molecules is beyond doubt. However, it is possible that targeting other proteins could have a similar impact. In fact, few overlap exists between the molecules targeted in each of these individual papers, especially those that used functional genomics to identify potential targets. This can be interpreted as to say that these papers have found "a set", (but not a unique "set") of targets that are therapeutically relevant. Finding a correct algorithm for the combination of drugs that target a set of proteins critical for sustaining cancer cells should be pursued.

In conclusion, recent results using new "omic" techniques support the future use of targeted drug combinations for the treatment of solid tumours. In our opinion to increase the efficiency of this process, it is critical to reach a close collaboration between academia, the pharmaceutical and biotechnological companies as well as regulatory authorities to develop rationale studies with drug combinations based on strong preclinical data.

## Competing interests

The authors declare that they have no competing interests.

## Authors' contributions

Both authors contributed equally to the writing of the paper. Both authors also read and approved the final manuscript.
